# A Vaccine Based on the A/ASIA/G-VII Lineage of Foot-and-Mouth Disease Virus Offers Low Levels of Protection against Circulating Viruses from the A/ASIA/Iran-05 lineage

**DOI:** 10.3390/v14010097

**Published:** 2022-01-06

**Authors:** Nagendrakumar Balasubramanian Singanallur, Phaedra Lydia Eblé, Anna Barbara Ludi, Bob Statham, Abdelghani Bin-Tarif, Donald P. King, Aldo Dekker, Wilna Vosloo

**Affiliations:** 1 Australian Centre for Disease Preparedness, CSIRO-Health & Biosecurity, 5 Portarlington Road, Geelong, VIC 3220, Australia; nagendra.singanallur@csiro.au; 2 Laboratory Vesicular Diseases, Department of Virology and Molecular Biology, Wageningen Bioveterinary Research, Houtribweg 39, 8221 RA Lelystad, The Netherlands; Phaedra.Eble@wur.nl (P.L.E.); Aldo.Dekker@wur.nl (A.D.); 3 The Pirbright Institute, Ash Road, Pirbright, Woking GU24 ONF, UK; anna.ludi@pirbright.ac.uk (A.B.L.); robert.statham@pirbright.ac.uk (B.S.); abid.bin-tarif@pirbright.ac.uk (A.B.-T.); donald.king@pirbright.ac.uk (D.P.K.)

**Keywords:** foot-and-mouth disease virus, vaccine efficacy, serotype A/ASIA/G-VII lineage vaccine, A/ASIA/Iran-05 lineage variant, heterologous challenge, cattle

## Abstract

The recent emergence and circulation of the A/ASIA/G-VII (A/G-VII) lineage of foot-and-mouth disease virus (FMDV) in the Middle East has resulted in the development of homologous vaccines to ensure susceptible animals are sufficiently protected against clinical disease. However, a second serotype A lineage called A/ASIA/Iran-05 (A/IRN/05) continues to circulate in the region and it is therefore imperative to ensure vaccine strains used will protect against both lineages. In addition, for FMDV vaccine banks that usually hold a limited number of strains, it is necessary to include strains with a broad antigenic coverage. To assess the cross protective ability of an A/G-VII emergency vaccine (formulated at 43 (95% CI 8–230) PD_50_/dose as determined during homologous challenge), we performed a heterologous potency test according to the European Pharmacopoeia design using a field isolate from the A/IRN/05 lineage as the challenge virus. The estimated heterologous potency in this study was 2.0 (95% CI 0.4–6.0) PD_50_/dose, which is below the minimum potency recommended by the World Organisation for Animal Health (OIE). Furthermore, the cross-reactive antibody titres against the heterologous challenge virus were poor (≤log_10_ 0.9), even in those cattle that had received the full dose of vaccine. The geometric mean r_1_-value was 0.2 (95% CI 0.03–0.8), similar to the potency ratio of 0.04 (95% CI 0.004–0.3). Vaccination decreased viraemia and virus excretion compared to the unvaccinated controls. Our results indicate that this A/G-VII vaccine does not provide sufficient protection against viruses belonging to the A/IRN/05 lineage and therefore the A/G-VII vaccine strain cannot replace the A/IRN/05 vaccine strain but could be considered an additional strain for use in vaccines and antigen banks.

## 1. Introduction

Foot-and-mouth disease (FMD) is a contagious disease of cloven-hoofed animals that can cause high morbidity in affected animals, but mortality is usually low. However, the mortality rates depend on the age of the animal, with young animals more likely to succumb to the disease. FMD is an economically important disease of livestock and may spread over large distances [1]. FMD is caused by FMD virus (FMDV; family Picornaviridae, genus Aphthovirus), which has seven immunologically distinct serotypes: O, A, C, Asia 1, SAT 1, SAT 2, and SAT 3, of which serotype C is likely extinct [2]). Based on ecologic niches of FMDV circulation, seven virus pools are defined, and Pools 1–3 are present in Asia, where only serotypes O, A, and Asia 1 are normally present [3,4,5].

Serotype A viruses show more genetic and antigenic variation and are grouped under three topotypes (ASIA, AFRICA, and EURO-SA (Europe-South America)) with multiple diverse lineages and sublineages [5]. The A/ASIA topotype is widely distributed in endemic countries in Asia and is also responsible for sporadic incursions into North Africa. One of the lineages within this topotype, A/ASIA/G-VII (A/G-VII; also referred to as genotype 18), originated from Pool 2 countries [6,7], and prior to 2015 only caused occasional and limited outbreaks in other regions: Saudi Arabia (1995), Albania and the former Yugoslav Republic of Macedonia (presently North Macedonia, 1996), and Myanmar (2010) [8].

During 2015, the A/G-VII lineage spread from Pool 2 to Saudi Arabia and Iran in the Middle East [9] and became established as one of the dominant strains after further spread into Armenia, Turkey, and northern Israel during 2017 [10,11,12]. The A/G-VII is an FMDV lineage that is genetically and antigenically discrete from A/ASIA/Iran-05 (A/IRN/05) FMDVs that are established in the Middle East. Across the P1 region, the A/G-VII and A/IRN/05 lineages exhibit approximately 85% nucleotide identity and 93% amino acid similarity (based on a comparison between prototype sequences A/IRN/27/2013 vs. A/IND40/2000 for A/ASIA/Iran-05^SIS^^−1^^3^ and A/ASIA/G-VII, respectively). Vaccine matching studies showed that isolates belonging to this lineage were antigenically heterologous to the commercial vaccine strains, A22/IRQ/64, A/Iran-96, A/Iran-05, and A/Sau-95 [13]. In addition, a study using a commercial polyvalent vaccine incorporating six different vaccine strains (A/IRN/05, A Saudi-95, O1 Manisa, O-3039, Asia-1 Shamir, and SAT-2 Eritrea) offered only partial protection (56% protection, i.e., approximately 1 PD_50_/dose) against A/IRN/10/2018, a virus belonging to the A/G-VII lineage [13]. A second pilot study indicated that the A/Malaysia-97 (A/MAY/97) vaccine strain provided better protection against challenge with this A/G-VII virus compared to A22/IRQ/64, and a subsequent full heterologous potency test with A/MAY/97 showed the vaccine had a potency of 6.5 PD_50_/dose [14].

The increased geographic distribution of the A/G-VII lineage and the poor antigen matching results with available FMD vaccines led vaccine manufacturers to develop new vaccines specific to this lineage. These new vaccines are expected to provide protection against field viruses from the A/G-VII lineage, but it is unknown whether these vaccines will provide broader protection to the endemic serotype A lineage (A/IRN/05) that co-circulates in the Middle East.

This study was undertaken to assess the heterologous potency of a A/G-VII vaccine with payload equivalent to 43 (95% CI 8–230) PD_50_/dose, in cattle against a representative field isolate belonging to the A/IRN/05 lineage. These data provide vital information to help understand whether the A/G-VII vaccine provides comprehensive coverage against circulating serotype A strains in West, Central, and South Asian countries with potential to replace existing vaccines used in endemic countries. These results also inform vaccine bank managers in FMD-free countries where there are cost and other considerations to maintain a broad range of vaccine antigens for emergency purposes.

## 2. Materials and Methods

### 2.1. Cells and Viruses Used in the Study

IBRS-2 (renal swine) cells were used for the virus neutralisation test (VNT). Primary lamb kidney cells were used for virus isolation. The challenge virus, A/IRN/10/2018, was originally isolated from bovine tongue epithelium from a cow infected with FMDV in 2018 in Iran and identified as belonging to the FMDV A/IRN/05 lineage (SIS-13 sub-lineage). The A/IRN22/2015 virus was also isolated from bovine tongue epithelium from a cow infected with FMDV in 2015 and was identified as a representative field isolate of the A/G-VII lineage. Both the virus isolates were provided by the World Reference Laboratory for FMD (WRLFMD), The Pirbright Institute, United Kingdom. The strain A/IRN/10/2018 was subsequently passed in a cow to prepare the cattle challenge virus used for the heterologous potency test at Wageningen Bioveterinary Research (WBVR), Lelystad, The Netherlands, as well for adaptation to IBRS-2 cells for VNTs.

### 2.2. Animal Ethics Committee Approvals

All the protocols for experimentation with live cattle were approved by the Australian Centre for Disease Preparedness (ACDP) Animal Ethics Committee (AEC 1926) and by the Institutional Animal Ethics Committee of WBVR (2016.D-0062.015 LVZ238). The trial was performed in the high containment animal facility of WBVR.

### 2.3. Vaccine, Potency Study, and Sample Collection

In total, 18 FMD seronegative dairy cattle (Holstein Friesian and mixed Holstein Friesian with local dairy cattle breeds) 8–12-months of age weighing 200–250 kg were used for the experiments. An experimental monovalent double oil emulsion vaccine with A/G-VII at an antigen payload that previously had shown a potency of 43 (95% CI 8–230) PD_50_/dose was prepared by Boehringer Ingelheim (BI), United Kingdom (homologous vaccine potency data shared by BI). A full heterologous potency was performed with three groups of five cattle vaccinated intramuscularly in the neck with either a full, 1/4, or 1/16 dose of vaccine (Supplementary Table S1) using standard protocols [15,16]. The vaccinated cattle, as well as three control cattle, were challenged 21 days post-vaccination (dpv).

Before challenge, all cattle were anaesthetized by administration of xylazine (1 mL per 100 kg, intravenously). The A/IRN/05 challenge virus (A/IRN/10/2018) was diluted in Minimum Essential Medium with Hanks’ balanced salts, 2% foetal bovine serum (FBS), and 2% antibiotic cocktail (the antibiotic cocktail contains 1000 IU/mL penicillin, 1000 µg/mL streptomycin, 20 µg/mL amphotericin B, 500 µg/mL polymyxin B, and 2400 µg/mL kanamycin sulphate) to give a final titre of 10^6.2^ PFU/mL. Each animal was challenged by the intra-dermo-lingual (IDL) route with 0.1 mL of the above virus dilution into two sites on the dorsum of the tongue (10^5.5^ PFU/0.2 mL), and this dose is equivalent to 10,000 cattle ID_50_ [17]. Atipamezole was used to reverse the anaesthetic (1 mL per 200 kg, intramuscularly). The cattle were observed daily post-vaccination and post-challenge. Flunixin meglumine 3.33 mg/kg body weight, equivalent to 1 mL/15 kg body weight (Finadyne pour-on 50 mg/mL, MSD Animal Health, The Netherlands) was given as a pre-emptive pain relief on the day of challenge, and repeated every second day in cattle with moderate or severe clinical signs. Lesions on the feet were considered generalisation of disease and, therefore, not protected [12]. Control cattle are expected to have lesions on at least three feet to ensure the challenge was sufficient. Mouth and nose lesions at sites other than the injection sites (dental pad, lips, etc.) were recorded but cattle with only mouth lesions were not considered unprotected. Rectal temperatures were recorded daily, starting on the day of challenge. The cattle were monitored daily for 8 days post-challenge (dpc) for clinical disease, with detailed examinations carried out on 4 dpc following sedation with xylazine (2 mg/kg) administered intramuscularly and on 8 dpc at post-mortem. All the cattle were humanely euthanized on 8 dpc using an overdose of sodium pentobarbital (Dechra Pharmaceuticals, Northwich, United Kingdom).

Clotted blood was collected on −21, −18, −14, −11, −7, −4, 0–8 dpc for serology. At 0–8 dpc, oral swabs were collected by inserting Salivette swabs (Sarstedt) in the mouth using forceps. In the laboratory the oral fluid was extracted by pipetting 1 mL of Minimum Essential Medium, 2% foetal bovine serum (FBS), and 10% antibiotic cocktail on the Salivette swabs and after mixing the swabs were allowed to absorb the medium for 15 min at room temperature. Following centrifugation for 10 min at 1780 g, the fluid was transferred to fresh tubes and stored at −70 °C until testing. Nasal swabs were collected on the same days using sterile cotton swabs. Two millilitres of Minimum Essential Medium, 2% FBS, and 10% antibiotic cocktail were added to the swab in the laboratory. After 15 min at room temperature the swab was removed and the fluid transferred to fresh tubes and stored at −70 °C until testing.

### 2.4. Serological Assays

Serum samples were examined for anti-FMDV neutralising antibodies using the VNT with IBRS-2 cells following standard procedures [16,18]. The VNT was performed using A/IRN/22/2015 (a representative field isolate from the A/G-VII lineage), as well as the heterologous challenge virus, A/IRN/10/2018 from the A/IRN/05 lineage. The neutralising antibody titres were calculated as the log_10_ of the reciprocal antibody dilution required for 50% neutralisation of 100 TCID_50_ virus (tests with a virus dose between 30 and 300 TCID_50_ were accepted as valid). Antibodies to the non-structural proteins (NSPs) of FMDV were detected using an ELISA where the serum samples were tested in duplicate, diluted at 1:5, following the standard operating protocol implemented at WBVR, using the PrioCHECK^®^ FMDV-NS kit (Thermofisher Scientific Waltham, MA, USA). Samples showing >50 per cent inhibition (PI) value were considered as positive.

### 2.5. Vaccine Matching of Field Isolates

Vaccine matching was undertaken for five FMDV field isolates from the A/IRN/05^SIS^^−1^^3^ lineage using a 2D-VNT with IB-RS-2 cells based on the original method and outlined in the OIE Manual of Diagnostic Tests and Vaccines for Terrestrial Animals [16]. These field isolates from Iran, Afghanistan, and Pakistan were tested as part of the WRLFMD remit as an OIE/FAO reference laboratory. Vaccine matching data were generated against a panel of three bovine vaccinated sera (BVS), raised against a monovalent A/IRN/05, A/A22, or A/G-VII antigen containing vaccines. The sera used in the test were collected 21 days after vaccination with a high potency vaccine (>6PD_50_/dose) and were pooled from five cattle. Briefly, the titres were calculated as the antibody dilution required to neutralise 50% of virus/cell mixtures at a virus dose of 100 TCID_50_ and presented as the reciprocal. The 100 TCID_50_ was obtained by using five virus doses spanning from 10 to 1000 TCID_50_; each of these virus doses were tested against a serial two-fold dilution of sera. Using linear regression, the neutralisation at 100 TCID_50_ was derived. The r_1_-values were calculated by taking the arithmetic mean of the field virus neutralisation titre and dividing it by the arithmetic mean of the vaccine virus neutralisation titre. A value less than 0.3 indicates a significant difference between the vaccine virus and the field virus. Each r_1_-value is based on at least two sets of individual results. Using the sera produced in the experiment, the r_1_-value of individual vaccinated cattle was calculated, as well as the r_2_-value using the sera of the control cattle collected 5, 6, 7, and 8 days after challenge.

### 2.6. Virus Isolation and Titration on Cell Culture

Primary lamb kidney cells in six-well plates (BioCoat, Corning Incorporated, Corning, NY, USA) were infected with the supernatant of the oral and nasal swabs and serum diluted tenfold in Minimum Essential Medium, 2% foetal bovine serum (FBS), and 2% antibiotic cocktail. Virus titration procedures were carried out using the plaque assay methods described elsewhere [19,20,21]. Plaques were visualized 24 or 48 h (based on microscopic size of the plaques) post-infection by amido black staining of fixed cells. The plaques were counted and titres were expressed as log_10_ PFU/mL.

### 2.7. Real-Time RT-PCR Assay for Detection of FMD Viral RNA

Total RNA from serum, nasal, and oral swabs was isolated using the MagNA Pure 96 DNA and Viral NA Large Volume kit on the MagNA Pure 96 system (Roche^®^ Life Science, Penzberg, Germany). In each run of 96 samples, one negative, one high positive, and one moderate positive sample were included as extraction controls. The RT-PCR was carried out as described by the manufacturer (Roche^®^) using the LightCycler RNA Amplification Kit Hybridisation Probes and LightCycler 480 (Roche^®^ Life Science) according to the protocol described elsewhere [22]. Samples were considered positive when the fluorescence signal rose above the background signal (crossing point determined automatically by the second derivative maximum method for quantification by the software supplied by Roche Life Sciences (Penzberg, Germany)).

### 2.8. Quantitative Analysis

The homologous and heterologous potency of the A/G-VII vaccine were calculated using the Spearman-Kärber method [23], and logistic regression was done using R [24]. Confidence intervals for the binomial models were calculated using the delta method [25]. In the logistic regression, results from the present experiment were added to a dataset with results of 60 potency tests in 912 cattle previously generated by WBVR as well as published data and experiment is used as additional explanatory variable. This way the slope of the dose response curve is estimated using all data and the position (the PD_50_) on the data of the experiment. Clinical protection based on count data was analysed using the two-sided Fischer exact test. For statistical analysis, VNT titres <0.6 were changed to 0.45. ANOVA was used to test the statistical differences between groups. If a statistical difference was found, a pairwise *t*-test (with Holm correction) was used to analyse differences between groups. Group means and standard deviations were calculated and expressed as Mean ± SD. Titres against A/IRN/12/2015 were compared for the experiment with homologous and heterologous challenge using logistic regression using the slope of previous experiments as offset in the model. Longitudinal data on virus titres from serum, oral, and nasal swabs were analysed using a linear mixed model, using the lme4 library in R [24], in which animal number was a random variable and dpc and group were possible explanatory variables. Using forward selection, the best model with the lowest AIC (Akaike’s Information Criterion) was chosen. In the linear mixed model, samples from which no virus could be isolated were assigned a log_10_ titre of 0, and samples in which no RT-PCR curve was detected were assigned a Ct of 45. The duration of excretion was calculated as the time between the first and last positive sample. Duration of virus and RNA detection was evaluated by normal linear regression.

## 3. Results and Discussion

### 3.1. Vaccine Matching Studies with Field Isolates

The results of the vaccine matching studies performed using the A/G-VII BVS and the other commercial vaccine strains (recommended for the region) on the A/IRN/05 lineage field isolates are presented in Table 1. For the five field isolates tested, these results indicate that there is poor antigenic match between the A/G-VII vaccine and the field isolates (r_1_-value < 0.3) and that the vaccine is unlikely to provide good protection. These isolates did not show any detectable neutralising antibody titres against A/G-VII bovine vaccinate sera.

### 3.2. Potency Test with A/G-VII Vaccine

After challenge, all three unvaccinated controls developed clinical signs of FMD and showed generalised disease by 4 dpc, with lesions on all four feet. Three of the five cattle that received a full dose, and one cattle each in the 1/4 and 1/16 dose groups were protected from clinical disease and generalisation (Supplementary Table S1). The estimated heterologous potency was 2 PD_50_/dose (CI: 0.4–6.0).

### 3.3. Quantitative Analysis of Serological Response to A/IRN/22/205 and A/IRN/18/2018

The serological responses to the vaccine were determined against the A/G-VII lineage and A/IRN/05 lineage FMD viruses. Prior to vaccination, all animals were seronegative to FMDV serotype A by VNT, with no demonstrable antibody titres to either the A/G-VII field isolate or the heterologous challenge virus A/IRN/10/2018.

**Figure 1:**
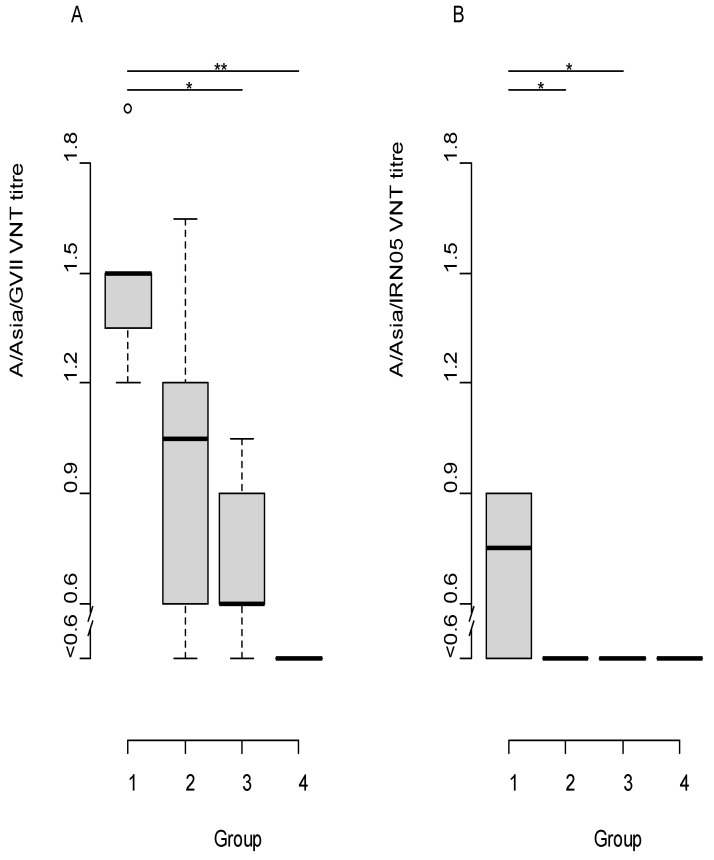
Box plot of VNT titres of cattle vaccinated with A/G-VII on the day of challenge (21 dpv). The horizontal line represents the median titre for each group. In panel (**A**) the VNT titres against A/IRN/22/2015 are shown (representative A/G-VII field isolate); in panel (**B**) the VNT titres against A/IRN/10/2018 are shown. Group 1 = Full dose, 2 = 1/4 dose, 3 = 1/16 dose, 4 = Unvaccinated Control); * = *p* < 0.05 and ** = *p* < 0.01. The boxplots show the interquartile range (median represented as the thick horizontal line within the box, and the first and third quartile of the data) and the minimum and maximum values for each group connected to the boxes with the vertical line.

Neutralising antibodies were observed around 7 dpv against the A/G-VII virus in two animals belonging to the full dose group and one animal in 1/4 dose group. By 14 dpv and 21 dpv (0 dpc), all animals that had received the full dose and only two of those that had received 1/4 dose had FMDV-specific antibodies. The animals that had received 1/16 dose had low A/G-VII-specific FMDV antibodies at the time of challenge (Supplementary Table S2). The mean antibody titres on the day of challenge (21 dpv) for the three vaccine groups were 1.5, 1.0, and 0.7 for the full dose, 1/4, and 1/16 groups, respectively. Only three cattle in the full dose group showed antibody titres above the detection limit against the heterologous challenge virus A/IRN/10/2018 by 21 dpv (Supplementary Table S3, Figure 1B).

The mean A/G-VII-specific VNT titres (Figure 1A) of the full dose group was significantly higher when compared to the 1/16 and unvaccinated groups and not significantly different when compared to the 1/4dose group (ANOVA *p* = 0.0001; pairwise ‘*t*’ test post hoc). The mean heterologous VNT titres were significantly different (ANOVA *p* = 0.02), the post hoc test using pairwise ‘*t*’ test showed significant difference between full and 1/4 dose as well as between full and 1/16 dose groups (Figure 1B).

**Figure 2:**
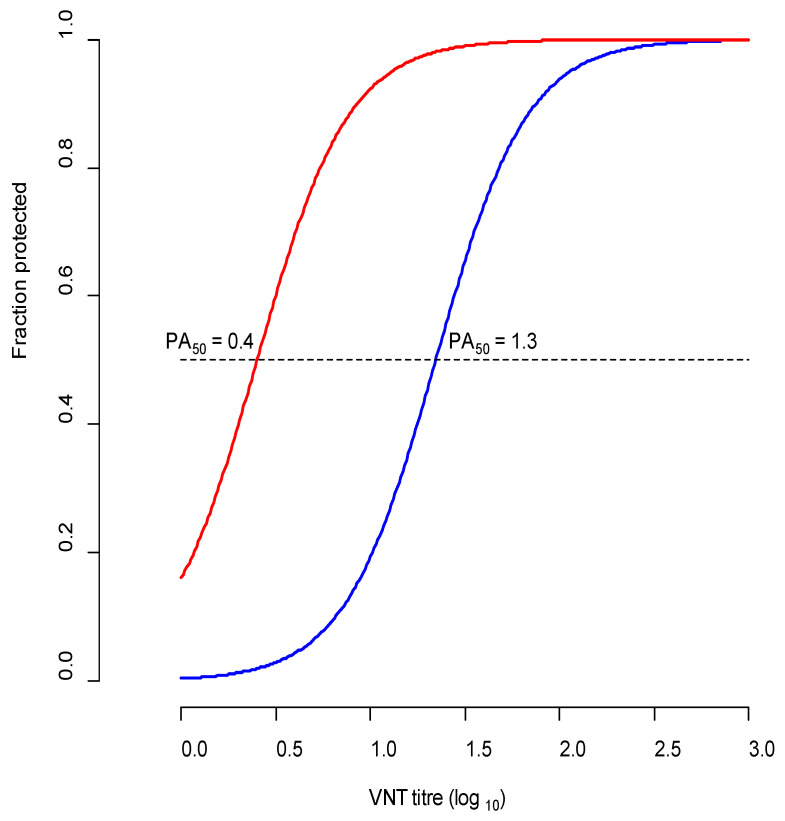
Logistic regression plot showing the relationship between VNT titres against A/IRN/22/2015 (representative A/G-VII field isolate) of cattle vaccinated with A/G-VII vaccine on the day of challenge (21 days post vaccination) and protection. The red line indicates protection after challenge with A/IRN/22/2015, and the blue line indicates protection after challenge with A/IRN/10/2018. Horizontal dotted line for 50 percent protection intersects the regression lines at the PA_50_ values for A/G-VII and A/IRN/05 protection.

Figure 2 shows the comparison of A/G-VII and A/IRN/05-specific VNT titres in relation to protection using logistical regression. The figure shows the titre that correlates with 50% protection (PA_50_). The PA_50_ for homologous protection was 0.4, whereas for heterologous protection with A/IRN/10/2018 the PA_50_ was 1.3. The estimated r_1_ values based on individual 21 dpv titres from the full vaccine group ranged between <0.18 and 0.25, indicating that there is poor serological match and the A/G-VII vaccine will not protect against clinical disease if challenged by A/IRN/05 lineage viruses.

All the cattle in the different groups seroconverted to the NSPs by 6 dpc (results not shown), indicating virus replication. This was expected as the cattle were infected by needle inoculation in the tongue. Because the potency tests are based on inoculating the virus on the dorsum of the tongue, primary virus replication occurs at the site of inoculation, leading to generation of NSPs and induction of anti-NSP antibodies. This local replication that often leads to minor lesions on the tongue is not considered vaccine failure; failure occurs only when secondary lesions appear on the limbs. In other challenge models where the virus is either instilled in the nasal passage (intranasal instillation) or by direct contact challenge using donor animals, sterile immunity is more often observed in vaccine trials.

### 3.4. Virus Isolation and Detection of Viral RNA in Clinical Samples

Virus was isolated from the serum of all control cattle between 1 and 3 dpc, and from one cow in the 1/4 dose group (2 dpc) and one cow in the 1/16 dose group (2–3 dpc) but not from the cattle in the full-dose group (Table 2). Viral RNA could be detected by real-time RT-PCR in the serum of the full dose group up to 3 dpc, and up to 5 dpc in the other two vaccine groups as well as the unvaccinated cattle (Table 2).

FMDV was isolated intermittently from the nasal swabs of some of the vaccinated cattle between 1 and 3 dpc (Table 2). This contrasts with the unvaccinated cattle that showed virus excretion more consistently for up to 5 dpc. Viral RNA was detected in nasal swabs between 1 and 4 dpc from all vaccinated cattle, with a decrease in the number of positive samples up to 7 dpc and consistently up to 8 dpc in the unvaccinated control cattle (Table 2).

Infectious virus was isolated and titrated from the oral swabs across all the vaccine groups between 1 and 5 dpc and up to 8 dpc in the unvaccinated control groups (Table 2). However, it should be noted that the cattle were infected via IDL challenge and therefore had lesions on their tongues. Viral RNA was detected on all days up to 8 dpc from the mouth swabs, but a decrease was noted in the full dose and 1/4 dose groups (Table 2).

Statistical analysis by forward regression in a linear mixed model showed a significant difference in virus as well as RNA levels in the different sample types, and significant differences between dpc. There was also a significant difference between the vaccine groups compared to the control group. Duration of virus and RNA detection depended on sample type and group, where the non-vaccinated controls showed the most significant difference with the full dose group. In protected cattle there was a significant difference in the duration of viral RNA excretion, mainly seen in the serum samples (statistically significant interaction between sample type and protection).

When compared with the full dose group, the other vaccine groups did not show any significant difference in virus levels in oral and nasal swabs, but the levels were significant when compared to the control group. This indicates that although the vaccine did not protect from clinical disease, it reduced viraemia and virus shedding in the oral and nasal secretions. These results concur with earlier studies [26], which concluded that the transmission between animals is based on infectivity, susceptibility, and contact rate. In the potency test we do not measure reduction of susceptibility, as all cattle develop tongue lesions, but reduction of infectivity. As generalisation will increase the contamination of the environment with virus, lower virus excretion decreases the chance for transmission. In the field reduction of excretion will have a significant influence on reduction of transmission [27,28].

## 4. Conclusions

The A/G-VII vaccine has a heterologous potency of 2 PD_50_/dose against the challenge virus A/IRN/22/2015 belonging to the A/IRN/05 lineage, which is below the OIE recommended minimum potency of 3 PD_50_/dose (https://www.oie.int/fileadmin/Home/eng/Health_standards/tahm/3.01.08_FMD.pdf; accessed on 1 October 2021). The true homologous potency of the vaccine in this trial is not known. However, based on the potency test results and serological data shared by the vaccine manufacturer with the authors and the results obtained from the current study, it is valid to assume that both the vaccines (one used for the previous homologous potency test and the one used in the current study) have similar homologous potency estimates (43 PD_50_/dose (95% CI 8–230)).

Commercial companies are not at liberty to declare many details regarding vaccine strains. We therefore used substitute viruses that, to the best of our knowledge, are related to the actual vaccine strain. The sequenced A-GVII strains from 2015 (the BI vaccine strain is also from 2015) showed over 98% nucleotide similarities across the 1D region [29], and therefore we do not expect much antigenic difference between the vaccine strain and A/IRN/22/2015 used in our study.

Despite the poor heterologous potency estimates in the current study, the vaccinated animals had a shorter duration of viraemia while virus and viral RNA were detected for shorter periods and at lower titres in nasal swabs compared to the control animals. Furthermore, there was a significant correlation between the A/G-VII and A/IRN/05-specific heterologous neutralising titres and protection in the vaccinated animals. This vaccine could therefore have some value in decreasing virus excretion during outbreaks; however, other control measures will be equally important and a strain with higher potency is required to control outbreaks caused by A/IRN/05 FMD viruses.

The use of r_1_ values when estimating homologous relationships is well established, but there is more uncertainty when using these in vitro tests for heterologous protection [30]. However, analysis of the in vitro r_1_-value (<0.18–0.25) and the potency ratio (0.26) in this study shows that the confidence intervals largely overlap with the geometric mean r_1_-value of 0.2 (95% CI 0.03–0.8) and the potency ratio of 0.04 (95% CI 0.004–0.3). Therefore, a well standardised r_1_-value determination is still valuable to estimate heterologous potency.

Vaccine strains selected for routine use as well as for antigen banks in FMDV free countries should preferably have broad antigenic coverage. The results from this study demonstrate that the A/G-VII vaccine strain cannot replace the A/IRN/05 vaccine strain, but could be considered an additional strain for use where the A/G-VII viruses occur as well as for antigen banks. We previously assessed the efficacy of A22 IRQ and A/MAY/97 vaccine strains to challenge with A/IRN/22/2015 from the A/G-VII lineage [13] demonstrating that A/MAY/97 provides protection against the A/G-VII lineage and therefore, if A/MAY/97 is already included in the antigen bank, replacement with the A/G-VII antigen is not necessary.

## Figures and Tables

**Table 1:** Antigenic match between a selection of antiserum raised against serotype A vaccines and representative A/ASIA/Iran/05^SIS−13^ field isolates. These data are reported as the log_10_ reciprocal of the heterologous neutralisation titres (titre) and serological vaccine matching values (r_1_-value); r_1_-values below 0.3 indicate a significant difference between the vaccine virus and the field isolate. Bovine Vaccinated Sera (BVS) for the vaccine strains compared were provided by Boehringer Ingelheim. * The isolates did not show any detectable neutralising antibody titres against A/G-VII bovine vaccinate sera and hence represented as <0.60 and, accordingly, the r_1_-values are represented as less than the expected value if the titres were 0.60.

Isolate	A/IRN/05 Vaccine		A/A22 Vaccine		A/G-VII Vaccine *	
	Titre	r_1_-Value	Titre	r_1_-Value	Titre	r_1_-Value
IRN/10/2018	1.77	0.48	1.92	0.45	<0.60	<0.07
AFG/50/2017	1.86	0.13	1.84	0.21	<0.60	<0.06
IRN/23/2018	1.38	0.19	1.80	0.33	<0.60	<0.07
PAK/1/2018	2.04	0.36	1.99	0.32	<0.60	<0.06
PAK/24/2019	2.16	0.49	2.17	0.48	<0.60	<0.08

**Table 2:** Virus isolation and real-time PCR results from serum, nasal secretions, and oral swabs. The cattle were vaccinated either with a full dose, 1/4 dose, or 1/16 dose of A/ASIA/G-VII vaccine and were challenged 21 days after vaccination with an A/IRN/05 strain. Protection: P—protected and NP—not protected; DPC—day post-challenge; − No virus detected; samples in the grey shaded cells were positive for virus genome by real-time RT-PCR; S—serum; N—nasal swab; O—saliva sample.

Groups	Animal ID	Protection	1 DPC			2 DPC			3 DPC			4 DPC			5 DPC			6 DPC			7 DPC			8 DPC		
			S	N	O	S	N	O	S	N	O	S	N	O	S	N	O	S	N	O	S	N	O	S	N	O
Full dose	2780	P	−	−	1.40	−	−	3.56	−	0.40	3.86	−	−	1.57	−	−	−	−	−	−	−	−	−	−	−	−
	2781	NP	−	−	3.74	−	−	5.45	−	0.40	2.98	−	−	1.63	−	−	0.70	−	−	−	−	−	−	−	−	−
	2782	P	−	−	5.16	−	−	6.02	−	−	6.35	−	−	5.40	−	−	2.05	−	−	−	−	−	−	−	−	−
	2783	NP	−	0.4	5.39	−	2.97	4.10	−	−	1.40	−	−	−	−	−	−	−	−	−	−	−	−	−	−	−
	2784	P	−	−	4.74	−	−	7.02	−	−	3.48	−	−	2.58	−	−	−	−	−	−	−	−	−	−	−	−
1/4 dose	2785	NP	−	−	3.47	−	−	2.2	−	−	1.83	−	−	2.77	−	−	1.65	−	−	−	−	−	−	−	−	−
	2786	NP	−	2.31	4.68	−	−	3.59	−	−	1.60	−	−	1.51	−	−	−	−	−	−	−		−	−	−	−
	2787	NP	−	1.10	4.96	−	−	6.41	−	−	5.62	−	−	4.97	−	−	−	−	−	−	−	−	−	−	−	−
	2788	NP	−	1.00	3.47	1.40	0.88	3.72	−	1.18	2.02	−	−	0.40	−	−	−	−	−	−	−	−	−	−	−	−
	2789	P	−	−	4.14	−	1.40	3.82	−	−	5.66	−	−	2.11	−	−	0.40	−	−	−	−	−	−	−	−	−
1/16 dose	2790	NP	−	−	5.41	1.44	0.40	4.99	0.40	−	2.44	−	−	2.44	−	−	−	−	−	−	−	−	−	−	−	−
	2791	NP	−	−	3.64	−	0.40	4.92	−	−	2.09	−	−	6.18	−	−	1.35	−	−	−	−	−	−	−	−	−
	2792	NP	−	0.88	5.61	−	−	4.00	−	−	3.27	−	−	2.11	−	−	−	−	−	−	−	−	−	−	−	−
	2793	NP	−	1.65	3.97	−	−	2.19	−	−	3.02	−	−	1.60	−	−	−	−	−	−	−	−	−	−	−	−
	2794	P	−	−	4.88	−	−	3.24	−	−	3.49	−	−	3.57	−	−	−	−	−	−	−	−	−	−	−	−
UV Controls	2795	NP	2.98	−	4.51	1.60	2.98	4.8	1.70	2.26	4.50	−	1.85	3.56	−	1.00	3.09	−	−	5.61	−	−	5.05	−	−	−
	2796	NP	2.82	−	5.60	2.80	3.03	4.18	1.48	1.54	2.28	−	−	5.67	−	−	1.40	−	−	1.30	−	−	0.70	−	−	−
	2797	NP	1.35	−	4.79	2.20	2.39	6.43	1.70	1.92	6.18	−	0.70	5.17	−	−	4.9	−	−	0.88	−	−	0.40	−	−	−

## Data Availability

All data are archived as per the CSIRO policies and guidelines.

## Supplementary Materials

The following supporting information can be downloaded at: https://www.mdpi.com/article/10.3390/v14010097/s1, Table S1: Animal groups, vaccination, challenge and clinical signs post challenge; Table S2: Serum antibody titres (log_10_) against A/IRN/22/2015 in cattle vaccinated with A/Asia/G-VII vaccine in a heterologous potency test with A/IRN/10/2018 challenge; Table S3: Heterologous serum antibody titres (log_10_) against A/IRN/10/2018 in cattle vaccinated with A/Asia/G-VII vaccine in a heterologous potency test with A/IRN/10/2018 challenge.
