# Supplementary material for: A Vaccine Based on the A/ASIA/G-VII Lineage of Foot-and-Mouth Disease Virus Offers Low Levels of Protection against Circulating Viruses from the A/ASIA/Iran-05 lineage
Source: Viruses. 2022 Jan 6;14(1):97. doi: 10.3390/v14010097 (PMC8781018; doi:10.3390/v14010097)
Supplement: Supplementary file 1 [file viruses-14-00097-s001.zip › viruses-1458666-supplementary.pdf]

A/Asia/GVII vaccine vs A/Asia/Iran-05 lineage – Supplementary Tables

Supplementary Table S1: Animal groups, vaccination, challenge and clinical signs post challenge. T = Tongue; M = Mouth and lips; N = Nostrils; RF = Right Fore-limb; LF = Left Fore-limb; RH = Right Hind-limb; LH = Left Hind-limb; BF = Both Fore-limb; BH = Both Hind-limb

Group		Animal ID	4 dpc	8 dpc	Status post-challenge
Full dose	Vaccination of cattle with 2 ml A/ASIA/G-VII monovalent oil adjuvant vaccine (full dose) and challenged 21 days later with cattle adapted A/IRN/10/2018	2780			Protected
		2781	N, LF, RF, LH, RH	T, N, LF, RF, LH, RH	Not Protected
		2782			Protected
		2783	RF, LH, RH	T, RF, LH, RH	Not Protected
		2784			Protected
1/4 dose	Vaccination of cattle with 0.5 ml A/ASIA/G-VII monovalent oil adjuvant vaccine (1/4 dose) and challenged 21 days later with cattle adapted A/IRN/10/2018	2785	LF, RF, LH, RH	LF, RF, LH, RH	Not Protected
		2786	LF, LH	LH	Not Protected
		2787	RH	T, M, RH	Not Protected
		2788	LF, LH, LH	LF, RF, LH, RH	Not Protected
		2789		T	Protected
1/16 dose	Vaccination of cattle with 0.125 ml A/ASIA/G-VII monovalent oil adjuvant vaccine (1/16 dose) and challenged 21 days later with cattle adapted A/IRN/10/2018	2790	LF, LH	T, M, LF, LH, RH	Not Protected
		2791	LF, LH, RH	T, LF, RF, LH, RH	Not Protected
		2792		T, M, LF	Not Protected
		2793		LH	Not Protected
		2794		T, N	Protected
UV Control	Unvaccinated cattle, challenged with cattle adapted A/IRN/10/2018	2795	N, LF, RF, LH, RH	T, LF, RF, LH, RH	Not Protected
		2796	N, LF, RF, LH, RH	T, N, LF, RF, LH, RH	Not Protected
		2797	N, LF, RF, LH, RH	T, LF, RF, LH, RH	Not Protected

Supplementary Table S2: Serum antibody titres (log_{10}) against A/IRN/22/2015 in cattle vaccinated with A/Asia/G-VII vaccine in a heterologous potency test with A/IRN/10/2018 challenge. The animals were vaccinated either with a full dose, one fourth dose or one sixteenth dose of A/Asia/G-VII vaccine and were challenged 21 days after vaccination (0 DPC). DPC = Day post challenge. - = titre <0.6

Groups	Animal ID	−21 DPC	−14 DPC	−7 DPC	0 DPC	1 DPC	2 DPC	3 DPC	4 DPC	5 DPC	6 DPC	7 DPC	8 DPC
Full dose	2780	<0.60	1.35	1.20	1.50	1.35	1.65	1.95	1.65	2.40	≥2.85	2.70	≥2.85
	2781	<0.60	1.35	1.50	1.35	1.50	1.20	1.65	1.80	2.55	≥2.85	≥2.85	≥2.85
	2782	<0.60	1.05	1.65	1.95	1.80	1.65	1.65	2.10	2.55	2.70	≥2.85	≥2.85
	2783	<0.60	1.05	1.35	1.20	1.20	1.80	1.50	2.25	≥2.85	≥2.85	≥2.85	≥2.85
	2784	<0.60	0.90	1.50	1.50	1.65	1.65	1.20	1.95	2.55	2.70	≥2.85	≥2.85
1/4 dose	2785	<0.60	<0.60	0.60	<0.60	0.60	0.60	0.90	1.20	1.65	2.55	2.55	≥2.85
	2786	<0.60	1.20	1.20	1.20	1.20	1.35	1.50	1.80	≥2.85	≥2.85	≥2.85	≥2.85
	2787	<0.60	0.75	0.90	0.60	0.90	0.75	1.05	1.65	2.40	2.70	≥2.85	≥2.85
	2788	<0.60	0.75	1.05	1.05	0.75	0.75	1.20	1.95	≥2.85	≥2.85	≥2.85	≥2.85
	2789	<0.60	1.05	1.50	1.65	1.50	1.65	1.65	2.10	2.55	≥2.85	≥2.85	≥2.85
1/16 dose	2790	<0.60	<0.60	0.60	<0.60	<0.60	0.75	0.75	1.20	2.40	≥2.85	≥2.85	≥2.85
	2791	<0.60	<0.60	0.90	0.60	0.75	0.90	0.75	1.95	2.70	≥2.85	≥2.85	≥2.85
	2792	<0.60	<0.60	0.75	1.05	1.20	1.05	1.05	1.80	≥2.85	≥2.85	≥2.85	≥2.85
	2793	<0.60	0.75	1.05	0.60	0.75	0.75	0.75	1.65	1.95	≥2.85	≥2.85	≥2.85
	2794	<0.60	<0.60	0.75	0.90	0.90	0.75	1.05	1.65	2.70	≥2.85	≥2.85	≥2.85
UV control	2795	<0.60	<0.60	<0.60	<0.60	<0.60	0.75	<0.60	<0.60	0.75	0.90	1.35	1.80
	2796	<0.60	<0.60	<0.60	<0.60	<0.60	0.75	<0.60	<0.60	1.05	1.35	1.65	1.65
	2797	<0.60	<0.60	<0.60	<0.60	<0.60	<0.60	<0.60	<0.60	0.60	1.20	1.50	1.80

Supplementary Table S3: Heterologous serum antibody titres (log_{10}) against A/IRN/10/2018 in cattle vaccinated with A/Asia/G-VII vaccine in a heterologous potency test with A/IRN/10/2018 challenge. The animals were vaccinated either with a full dose, one fourth dose or one sixteenth dose of A/Asia/G-VII vaccine and were challenged 21 days after vaccination (0 DPC). DPC = Day post challenge. - = titre <0.6

Groups	Animal ID	−21 DPC	−14 DPC	−7 DPC	0 DPC	1 DPC	2 DPC	3 DPC	4 DPC	5 DPC	6 DPC	7 DPC	8 DPC
Full dose	2780	<0.60	1.05	<0.60	0.90	0.75	0.75	1.05	1.20	2.55	2.70	2.55	≥2.85
	2781	<0.60	<0.60	<0.60	<0.60	<0.60	<0.60	0.75	1.65	2.40	≥2.85	≥2.85	≥2.85
	2782	<0.60	0.60	0.60	0.75	0.90	0.90	1.20	1.35	2.40	2.55	2.70	≥2.85
	2783	<0.60	0.60	<0.60	<0.60	<0.60	<0.60	0.90	1.95	2.55	≥2.85	≥2.85	≥2.85
	2784	<0.60	0.75	0.75	0.90	0.75	1.05	1.05	1.05	2.10	2.55	2.70	≥2.85
1/4 dose	2785	<0.60	0.60	<0.60	<0.60	<0.60	<0.60	<0.60	1.05	1.80	2.70	2.70	≥2.85
	2786	<0.60	<0.60	0.60	<0.60	<0.60	<0.60	1.35	1.50	2.70	≥2.85	≥2.85	≥2.85
	2787	<0.60	<0.60	<0.60	<0.60	<0.60	<0.60	1.05	1.50	2.70	≥2.85	≥2.85	≥2.85
	2788	<0.60	<0.60	<0.60	<0.60	<0.60	<0.60	1.05	1.95	2.70	≥2.85	≥2.85	≥2.85
	2789	<0.60	<0.60	0.60	<0.60	0.60	0.90	0.75	1.35	2.25	≥2.85	≥2.85	≥2.85
1/16 dose	2790	<0.60	<0.60	<0.60	<0.60	<0.60	<0.60	0.60	1.20	2.70	2.70	≥2.85	≥2.85
	2791	<0.60	<0.60	<0.60	<0.60	<0.60	0.75	0.90	1.80	2.70	≥2.85	≥2.85	≥2.85
	2792	<0.60	<0.60	<0.60	0.60	<0.60	0.75	0.60	1.50	≥2.85	≥2.85	≥2.85	≥2.85
	2793	<0.60	<0.60	<0.60	<0.60	<0.60	0.75	<0.60	1.50	2.70	2.70	≥2.85	≥2.85
	2794	<0.60	<0.60	<0.60	<0.60	<0.60	<0.60	<0.60	1.20	2.55	2.70	≥2.85	≥2.85
UV control	2795	<0.60	<0.60	<0.60	<0.60	<0.60	1.05	0.75	1.05	1.95	2.40	2.70	2.70
	2796	<0.60	<0.60	<0.60	<0.60	<0.60	1.05	0.60	1.35	2.10	2.40	2.70	2.70
	2797	<0.60	<0.60	<0.60	<0.60	<0.60	<0.60	0.60	0.90	1.95	2.25	≥2.85	≥2.85
